# Association Between Dystonia-Related Genetic Loci and Parkinson's Disease in Eastern China

**DOI:** 10.3389/fneur.2021.711050

**Published:** 2022-02-22

**Authors:** Wen-Yi Yang, Si-Si Jiang, Jia-Li Pu, Chong-Yao Jin, Ting Gao, Ran Zheng, Jun Tian, Bao-Rong Zhang

**Affiliations:** Department of Neurology, Second Affiliated Hospital, College of Medicine, Zhejiang University, Hangzhou, China

**Keywords:** Parkinson's disease, dystonia, single nucleotide polymorphism, *ARSG*, *NALCN*

## Abstract

**Background:**

Parkinson's disease (PD) and dystonia are closely related in terms of pathophysiology and clinical manifestations, but their common genetic characteristics remain unclear. Some genome-wide association studies (GWASs) and replication studies have revealed correlations between single nucleotide polymorphisms (SNPs) of the *ARSG, BDNF, NALCN, OR4X2, KIAA1715, and OR4B1* genes and dystonia. This study was conducted to assess the association between these genetic loci and PD in a population from Eastern China.

**Methods:**

We genotyped the SNPs (rs11655081 of *ARSG*; rs6265 of *BDNF*; rs61973742, rs1338051, rs9518384, and rs9518385 of *NALCN*; rs67863238 of *OR4X2*; rs10930717 of *KIAA1715*; and rs35875350 of *OR4B1*) in a cohort of 474 patients with PD and 439 healthy controls from East China. To determine the genotypes of these SNPs, we used an Agena MassARRAY Typer 4.0. Odds ratios (*OR*s) and 95% *CI*s were computed to evaluate the correlations between these SNPs and the risk of PD.

**Results:**

There were significant differences in the genotype distribution (*OR* = 0.649, 95% *CI* = 0.478–0.880) and minor allele frequency (MAF) (*OR* = 0.703, 95% *CI* = 0.533–0.929) of SNP rs61973742 (*NALCN*) between patients with PD and healthy controls. A significant difference was detected in the genotype distribution of rs11655081 (*ARSG*) (*OR* = 1.486, 95% *CI* = 1.080–2.045).

**Conclusion:**

Single nucleotide polymorphisms rs11655081 (*ARSG*) and rs61973742 (*NALCN*) may be associated with PD. The C allele of rs11655081 may increase the risk of PD, whereas the G allele of rs61973742 may be a protective factor.

## Introduction

Parkinson's disease (PD) is the second most common neurodegenerative disease, affecting ~2–3% of the population aged ≥65 years old (1). The clinical characteristics of PD are described as parkinsonism (classical motor features, such as bradykinesia, muscular rigidity, rest tremor, and postural imbalance) and non-motor features, such as emotional disorders, sleep disorders, and autonomic dysfunction (2). The cause of PD is still not clear, but genetic and environmental factors have been shown to work together in its risk (3). Among these genetic factors, increasing SNP loci were found to be related to the onset of PD in genome-wide association studies (GWASs) (4–6). Dystonia is a neurological condition characterized by involuntary movements or postures due to persistent or intermittent muscle contractions (7). Dystonia can be found both in isolation and in combination with other movement disorders (e.g., dystonia with parkinsonism and myoclonus) (8). Dystonia may occur in idiopathic PD and is more frequently seen as a complication in the treatment of PD (9). The etiology of dystonia remains unknown. However, increasing evidence suggests that genetic variants may function in dystonia, and *DYT* genes are usually the cause of monogenic dystonia (7). Moreover, previous studies have indicated that dystonia and PD may share some common genetic risk factors. For example, parkinsonism is generally detected with mutations in *DYT* genes (such as *DYT/PARK-TAF1, DYT5/PARK- GCH1, DYT/PARK-ATP1A3, and DYT/PARK-PRKRA*) (10). On the other hand, patients with PD having mutations in *PARK1, PARK6, PARK7*, and *PARK8* appear to often manifest dystonia (3).

Some SNP loci in genes, such as *TOR1A* and *GCH1* may increase the genetic burden in dystonia (11, 12). Among these SNPs, *BDNF* rs6265 was identified to be probably related to many diseases, such as PD (13). Recent studies have revealed more SNPs that may be related to the onset of dystonia. Two GWASs have been used to identify gene alterations that may predispose to dystonia (14). In the GWAS executed by Lohmann et al., Arylsulfatase G (ARSG) was found to play a role in dystonia (15). According to the GWAS conducted by Mok et al., the most statistically significant variants in dystonia included *NALCN* (rs61973742, rs1338051, rs9518385, and rs9518384), *OR4X2* (rs67863238), *KIAA1715* (rs10930717), *and OR4B1* (rs35875350) (16). Among these genes, *NALCA* has been found to be associated with PD (17), while others have hardly been studied in patients with PD. To explore the genetic associations between these SNPs and PD, we performed a case-control study.

## Materials and Methods

### Subjects

Our study included 474 patients with PD without dystonia from the Neurology Department at the Second Affiliated Hospital of Zhejiang University. Patients with PD caused by secondary causes of Parkinsonism were excluded. Patients with other neurodegenerative diseases, such as essential tremor (ET), multiple system atrophy (MSA), corticobasal degeneration (CBD), and Wilson's disease (WD) were excluded. A total of 439 healthy individuals were recruited as the healthy controls. Both the patients and healthy controls were Han people from Eastern China.

Ethical approval was obtained from the Medical Ethics Committee of the Second Affiliated Hospital of Zhejiang University School of Medicine. Informed consent was obtained from all patients and healthy controls prior to blood withdrawal.

### Genotyping

Peripheral blood samples were collected from each patient. A DNA rapid extraction kit (BioTeKe Corporation, China) was used to extract DNA. AssayDesigner 3.1 (San Diego, CA: Agena Corporation) was used to design the genotyping assays. The following nine loci were tested: rs11655081 of *ARSG*; rs6265 of *BDNF*; rs61973742, rs1338051, rs9518384, and rs9518385 of *NALCN*; rs67863238 of *OR4X2*; rs10930717 of *KIAA1715*, and rs35875350 of *OR4B1*. Agena MassARRAY Typer 4.0 (San Diego, CA: Agena Corporation) was used to determine the genotypes of these SNPs.

### Statistical Analysis

The *t*-test was used to assess differences in age between patients with PD and controls. The chi-square test was used to detect the sex distribution and Hardy–Weinberg equilibrium (HWE) in healthy controls. Allelic, minor allele dominant, and recessive models were applied, and association analyses were performed using the chi-square test or Fisher's exact test. The odds ratio (*OR*) was used to assess the strength of polymorphisms and PD susceptibility. The Cochran-Armitage trend test (CATT) was used to calculate the additive model. All statistical analyses were performed using SPSS version 25.0 (Armonk, NY: IBM Corporation), and statistical significance was set at *p* < 0.05. The power was computed using the Quanto version 1.2.4.

## Results

A total of 474 patients with PD and 439 healthy controls were included. There were no significant differences in sex or age distribution between patients and controls (Table 1). High genotyping quality was achieved in all genotyping reactions. No significant deviation from the HWE was detected in the genotype frequency distributions of controls (Table 2). There were significant differences in the genotypes and allele frequencies of SNP rs61973742 (*NALCN*) between patients with PD and controls, as shown in Tables 2, 3. Using allelic, additive, minor allele dominant, and recessive models, we found that the minor allele frequency (MAF) of rs61973742 in PD cases was 10.81%, which was lower than that in healthy controls (*p* = 0.014, *OR* = 0.703, 95% *CI* = 0.533–0.929), and a difference in genotype distribution was observed in the additive (*p* = 0.011) and dominant models (*p* = 0.005, *OR* = 0.649, 95% *CI* = 0.478–0.880). The results also demonstrated an increase in the frequency of the CC genotype (recessive model) for SNP *ARSG* rs11655081 in patients with PD compared with healthy controls (*p* = 0.015, *OR* = 1.486, 95% *CI* = 1.080–2.045). The power of the recessive model in rs11655081 reached nearly 70% and the power of the dominant model of rs6197374 was nearly 80% (Table 4). There were no significant differences in the allele frequencies or genotype distributions of the SNP rs6265 of *BDNF*; rs1338051, rs9518384, and rs9518385 of *NALCN*; rs67863238 of *OR4X2*; rs10930717 of *KIAA1715*; and rs35875350 of *OR4B1* between patients and controls. The MAF in our population was consistent with 1,000 Genomes in PubMed (Supplementary Table 1).

**Table 1:** Demographic characteristics of patients with Parkinson's disease (PD) and healthy controls.

	**Cases (*n* = 474)**	**Controls (*n* = 439)**	***p*-value**
Sex (F/M)	201/273	174/265	0.359
Age (mean ± SD)	57.32 ± 11.74	57.63 ± 9.41	0.658
Age of onset (mean ± SD)	55.61 ± 9.79	N/A	N/A

**Table 2:** Allele frequency of patients with PD and healthy controls.

**Gene**	**SNP**	**HWE**	**Alleles**	**MAF (%)**		***p*-value**	**OR (95%CI)**
				**Cases**	**Controls**		
*ARSG*	rs11655081	0.086	T > C	464 (50.00%)	395 (45.51%)	0.057	1.197 (0.995–1.442)
*BDNF*	rs6265	0.494	T > C	471 (48.89%)	412 (47.36%)	0.280	1.107 (0.921–1.331)
***NALCN***	**rs61973742**	0.088	A > G	102 (10.81%)	129 (14.69%)	**0.014**	**0.703 (0.533–0.929)**
	rs1338051	0.215	A > G	379 (39.98%)	359 (41.08%)	0.634	0.956 (0.792–1.152)
	rs9518384	0.234	T > C	377 (40.11%)	362 (41.42%)	0.570	0.947 (0.785–1.142)
	rs9518385	0.209	C > A	379 (40.06%)	362 (41.23%)	0.679	0.953 (0.790–1.149)
*OR4X2*	rs67863238	0.185	G > C	61 (6.45%)	51 (5.81%)	0.570	1.118 (0.761–1.641)
*KIAA1715*	rs10930717	0.381	G > C	261 (27.53%)	227 (25.97%)	0.453	1.083 (0.880–1.333)
*OR4B1*	rs35875350	0.128	G > A	61 (6.43%)	48 (5.57%)	0.352	0.302 (0.031–2.911)

*The positive locus is marked in bold font*.

**Table 3:** Genotype distributions between patients and controls.

**Gene**	**SNP**	**Additive**	**Dominant model**		**Recessive model**	
		***p*-value**	***p*-value**	**OR (95% CI)**	***p*-value**	**OR (95% CI)**
***ARSG***	**rs11655081**	0.053	0.452	1.121 (0.833–1.507)	**0.015**	**1.486 (1.080–2.045)**
*BDNF*	rs6265	0.264	0.140	1.255 (0.928–1.698)	0.758	1.015 (0.767–1.439)
***NALCN***	**rs61973742**	**0.011**	**0.005**	**0.649 (0.478–0.880)**	0.855	1.118 (0.339–3.688)
	rs1338051	0.645	0.615	0.933 (0.713–1.222)	0.818	0.961 (0.685–1.348)
	rs9518384	0.583	0.498	0.911 (0.695–1.193)	0.861	0.970 (0.693–1.359)
	rs9518385	0.623	0.608	0.932 (0.712–1.220)	0.786	0.954 (0.681–1.337)
*OR4X2*	rs67863238	0.572	0.414	1.183 (0.790–1.772)	0.308	0.310 (0.032–2.971)
*KIAA1715*	rs10930717	0.460	0.422	1.113 (0.857–1.445)	0.793	1.067 (0.657–1.734)
*OR4B1*	rs35875350	0.442	0.298	1.243 (0.824–1.874)	0.352	0.302 (0.031–2.911)

*The positive locus is marked in bold font*.

**Table 4:** The power of dominant and recessive models.

**Gene**	**SNP**	**MAF**	**Case**	**Control**	***N***	**CON per case**	**Dominant model**		**Recessive model**	
							**OR**	**Power**	**OR**	**Power**
*ARSG*	rs11655081	0.478	464	434	898	0.935	1.121	0.119	1.486	0.696
*BDNF*	rs6265	0.487	472	435	907	0.922	1.255	0.325	1.015	0.051
*NALCN*	rs61973742	0.127	472	439	911	0.930	0.649	0.790	1.118	0.055
	rs1338051	0.405	474	437	911	0.922	0.933	0.079	0.961	0.056
	rs9518384	0.407	473	439	912	0.928	0.911	0.103	0.970	0.053
	rs9518385	0.406	470	437	907	0.930	0.932	0.080	0.954	0.058
*OR4X2*	rs67863238	0.061	473	439	912	0.928	1.183	0.130	0.310	0.175
*KIAA1715*	rs10930717	0.268	474	437	911	0.922	1.113	0.127	1.067	0.057
*OR4B1*	rs35875350	0.060	474	431	905	0.909	1.243	0.181	0.302	0.173

*CON, controls*.

## Discussion

This study was conducted to assess the association of nine dystonia-related genetic loci in a Chinese population of patients with PD. We found that rs11655081 of *ARSG* and rs61973742 of *NALCN* were significantly correlated with PD. The C allele of rs11655081 may increase the susceptibility to PD, while the G allele of rs61973742 may decrease it.

The proteins encoded by *ARSG* hydrolyze sulfate esters; therefore, they participate in cell signaling, protein degradation, and hormone biosynthesis (18, 19). GWAS by Lohmann et al. suggested that rs11655081 of *ARSG* was related to the musician's dystonia and writer's cramp (15). A study in a Dutch patient group with musician's dystonia and writer's cramp found an accumulation of rare single nucleotide variants in the coding region of *ARSG*, which indicated that *ARSG* may function in the task-induced focal dystonia (20). A cohort containing 206 blepharospasm (BSP) patients and 206 controls from Greek was sequenced, and a subtle trend for the correlation of rs11655081 and BSP susceptibility was found (*p* = 0.088, *OR* = 0.64, 95% *CI* = 0.38–1.07) (21). No studies evaluating the relationship between rs11655081 and PD have been conducted.

According to a GWAS performed by Mok et al. in a British cohort with cervical dystonia (CD), the cluster of variants of *NALCN* was nearest to the genome-wide significance threshold. A replication case-control study in a Spanish population did not report any association between CD and *NALCN* rs61973742 (*p* = 0.1155, *OR* = 0.7098) (22). A study by Zhou et al. (23) in a Chinese population did not suggest that the SNP rs61973742 of *NALCN* plays a role in focal CD. However, this SNP showed a potential effect in the same direction as the previously reported GWAS. *NALCN* encodes a protein associated with sodium leak channels, and it is predominantly expressed in the nervous system and is involved in regulating the resting membrane potential and neuronal excitability. Previous studies found that the protein encoded by *the ANO3 (DYT24)* gene, which functions as calcium-activated chloride channels, can participate in the modulation of neuronal excitability, and its mutations have been identified in sporadic CD (24). Based on this, Mok et al. (16) assumed that *NALCN* may be a candidate gene for dystonia. Few animal studies found that akinesia and freezing were the main phenotypes of *NALCN*-deficient strains; therefore, they put forward a hypothesis that the *NALCN* may be involved in movement disorders clinically characterized by akinesia and freezing gait in humans, such as PD (25, 26). Moreover, previous studies suggested *that NALCA* may contribute to both disordered movement and psychiatric features in PD through gene-gene interactions (17). The specific function of our SNP loci in *NALCN* remains unknown.

The *BDNF* gene has been found to influence synaptic plasticity, and it has been indicated to play a role in a wide range of neurological diseases and injuries (27). An increasing number of studies have focused on the relationship between the risk of PD and *BDNF* SNPs, especially rs6265, although strong evidence is yet to be presented (28). Michałowska et al. (29) identified that the variation of rs6265 increased the risk of PD. However, Pal et al. (28) and Svetel et al. (30) found that the allele frequency and genotype distribution in patients with PD and healthy controls did not deviate significantly. Our results are consistent with these latter findings. To date, studies concerning the role of rs6265 in dystonia have yielded contradictory results (14, 31–33). Therefore, the role *of BDNF* in both PD and dystonia requires further exploration.

Our study aimed to examine the associations between SNPs in the selected genes and PD in an Eastern Chinese population. There were some limitations to our study. First, the clinical manifestation of PD is diverse; therefore, it would be better to set subtypes, such as early- or late-onset PD, and PD patients with or without dystonia, according to the potential function of the SNPs. In addition, new association studies have been performed. For example, SNPs in *COL8A1, DENND1A*, and *GABBR2* were found to be associated with cervical dystonia in a multicenter GWAS (5). These loci have not been replicated in case-control studies. Given the racial heterogeneity and limited sample size, more replication studies are needed to assess whether these loci and variants are common genetic factors between PD and dystonia.

## Conclusion

In this study, we found that rs11655081 and rs61973742 may be associated with PD. The C allele of rs11655081 may increase the risk, and the G allele of rs61973742 may be a protective factor. No significant differences were detected either in allele frequencies or genotype distributions in the SNP rs6265 of *BDNF*; rs1338051, rs9518384, and rs9518385 of *NALCN*; rs67863238 of *OR4X2*; rs10930717 of *KIAA1715*; and rs35875350 of *OR4B1* between patients and controls.

## Data Availability Statement

All data generated or used during the study are available from the corresponding author by request.

## Ethics Statement

The studies involving human participants were reviewed and approved by Medical Ethics Committee of the Second Affiliated Hospital of Zhejiang University School of Medicine. The patients/participants provided their written informed consent to participate in this study.

## Author Contributions

JT contributed to conception and design of the study. C-YJ and RZ organized the database. W-YY, TG, and RZ performed the statistical analysis. W-YY and S-SJ wrote the first draft of the manuscript. All authors contributed to manuscript revision, read, and approved the submitted version.

## Funding

This study was funded by the National Natural Science Foundation of China (Grant Numbers 81771216 and 81520108010) and the Key R&D Program of Zhejiang Province (Grant Number 2020C03020).

## Conflict of Interest

The authors declare that the research was conducted in the absence of any commercial or financial relationships that could be construed as a potential conflict of interest.

## Publisher's Note

All claims expressed in this article are solely those of the authors and do not necessarily represent those of their affiliated organizations, or those of the publisher, the editors and the reviewers. Any product that may be evaluated in this article, or claim that may be made by its manufacturer, is not guaranteed or endorsed by the publisher.
