# Supplementary material for: Association Between Dystonia-Related Genetic Loci and Parkinson's Disease in Eastern China
Source: Front Neurol. 2022 Feb 22;12:711050. doi: 10.3389/fneur.2021.711050 (PMC8901603; doi:10.3389/fneur.2021.711050)
Supplement: Supplementary file 1 [file Table_1.docx]

**Supplement Data**

**Supplement Table** MAF observed in 1000 Genomes from PubMed

| Gene | SNP | Alleles | Observed | 1000-G | 1000-GEA |
| --- | --- | --- | --- | --- | --- |
|  |  |  |  |  |  |
| *ARSG* | rs11655081 | T>C | 0.478 | 0.302 | 0.469 |
| *BDNF* | rs6265 | T>C | 0.487 | 0.201 | 0.488 |
| *NALCN* | rs61973742 | A>G | 0.127 | 0.150 | 0.137 |
|  | rs1338051 | A>G | 0.405 | 0.579 | 0.418 |
|  | rs9518384 | T>C | 0.407 | 0.579 | 0.417 |
|  | rs9518385 | C>A | 0.406 | 0.568 | 0.415 |
| *OR4X2* | rs67863238 | G>C | 0.061 | 0.069 | 0.061 |
| *KIAA1715* | rs10930717 | G>C | 0.268 | 0.096 | 0.283 |
| *OR4B1* | rs35875350 | G>A | 0.060 | 0.070 | 0.061 |

Abbreviations: MAF, observed in this study; 1000-G, MAF in 1000 Genomes from PubMed; 1000-GEA, MAF in 1000 Genomes of East Asian populations from PubMed.

The data from PubMed were collected on July 18, 2020.
